# *Azoarcus* sp. CIB, an Anaerobic Biodegrader of Aromatic Compounds Shows an Endophytic Lifestyle

**DOI:** 10.1371/journal.pone.0110771

**Published:** 2014-10-23

**Authors:** Helga Fernández, Nicolás Prandoni, Mercedes Fernández-Pascual, Susana Fajardo, César Morcillo, Eduardo Díaz, Manuel Carmona

**Affiliations:** 1 Department of Environmental Biology, Centro de Investigaciones Biológicas-CSIC, Madrid, Spain; 2 Plant Protection Department, Instituto de Ciencias Agrarias-CSIC, Madrid, Spain; Shanghai Jiao Tong University, China

## Abstract

**Background:**

Endophytic bacteria that have plant growth promoting traits are of great interest in green biotechnology. The previous thought that the *Azoarcus* genus comprises bacteria that fit into one of two major eco-physiological groups, either free-living anaerobic biodegraders of aromatic compounds or obligate endophytes unable to degrade aromatics under anaerobic conditions, is revisited here.

**Methodology/Principal Findings:**

Light, confocal and electron microscopy reveal that *Azoarcus* sp. CIB, a facultative anaerobe β-proteobacterium able to degrade aromatic hydrocarbons under anoxic conditions, is also able to colonize the intercellular spaces of the rice roots. In addition, the strain CIB displays plant growth promoting traits such nitrogen fixation, uptake of insoluble phosphorus and production of indoleacetic acid. Therefore, this work demonstrates by the first time that a free-living bacterium able to degrade aromatic compounds under aerobic and anoxic conditions can share also an endophytic lifestyle. The phylogenetic analyses based on the 16S rDNA and *nifH* genes confirmed that obligate endophytes of the *Azoarcus* genus and facultative endophytes, such as *Azoarcus* sp. CIB, locate into different evolutionary branches.

**Conclusions/Significance:**

This is the first report of a bacterium, *Azoarcus* sp. CIB, able to degrade anaerobically a significant number of aromatic compounds, some of them of great environmental concern, and to colonize the rice as a facultative endophyte. Thus, *Azoarcus* sp. CIB becomes a suitable candidate for a more sustainable agricultural practice and phytoremediation technology.

## Introduction

Rice (*Oryza sativa* L.) is probably the most important cereal crop in the world, feeding more than 50% of worlds population [1]. To feed the increasing population in a sustainable manner without the utilization of chemical fertilizers or pesticides it will be necessary the application of green biotechnologies. Thus, endophytic bacteria that have plant growth promoting characteristics are crucial in this endeavour. The so-called “true endophytes” spend most of their life cycle inside plant tissues without causing symptoms of plant damage [2], but some endophytes are able to live outside of the plant without losing the capacity to colonize disinfected seedlings [3]. Some endophytic bacteria exhibit beneficial effects on the host plant, such as plant growth promotion, the induction of increased resistance to pathogens, as well as the supply of fixed nitrogen to the host plant [4]. In addition some endophytes contribute to enhanced biodegradation of environmental soil pollutants [5], [6], and it has been described that endophytic bacteria equipped with the appropriate degradation pathways improve *in planta* degradation of aromatic hydrocarbons [7], [8].

Various kinds of endophytic bacteria have been found inside rice plants [4], including nitrogen-fixing grass-associated diazotrophs bacteria from the *Azoarcus* genus [9]. Thus, *Azoarcus communis* strain SWub3 [10], *Azoarcus indigens* strain VB32 [10] or the well-characterized *Azoarcus* sp. strain BH72 invade roots of Kallar grass and rice, living as endophytic bacteria [11]. However, the ecological distribution of the *Azoarcus* genus is more widespread, and many strains are free-living bacteria that participate in the biogeochemical cycling of large number of metabolites, both organic and inorganic, such as arsenic acid, hydrogen or aromatic compounds [12]–[15]. A phylogenetic analysis of the 16S rDNA sequences from *Azoarcus* species known so far shows a tree with two main branches (Figure S1), [9], [13], [16]. One of the branches includes the free-living bacteria that usually are inhabitants of waters and soils; many strains of this group have been described and/or isolated by their ability to degrade aromatic compounds at anoxic conditions, e.g., *Azoarcus/“Aromatoleum”* strain EbN1 [17] and *Azoarcus evansii* KB740 [18], [19]. In the other branch of the phylogenetic tree are located the endophytes such as *Azoarcus* sp. strain BH72 [11]. Interestingly, the group of free-living *Azoarcus* strains that are anaerobic biodegraders was suggested to be unable to interact with plants [9], [20], and they only received particular attention for their degradation and biotransformation abilities [21]–[23].

*Azoarcus* sp. CIB is a previously described bacterium with the ability to degrade under aerobic and/or anaerobic conditions a high number of aromatic compounds, including some toxic hydrocarbons such as toluene and *m*-xylene [14], [21], [24]. The results presented on this work demonstrate that *Azoarcus* sp. CIB has also the ability to grow in association with plants, colonizing the intercellular spaces of the exodermis of rice roots. In addition, the strain CIB displays plant growth promoting (PGP) traits. Therefore, this work demonstrates by the first time that a free-living bacterium able to degrade aromatic compounds in aerobic and anoxic conditions can share also an endophytic lifestyle.

## Materials and Methods

### Bacterial strains, seeds, and plasmids used

*Azoarcus* sp. strain CIB [14] is deposited in the Spanish Type Culture Collection (CECT#5669). *Azoarcus communis* strain SWub3 [25] was obtained from the German Collection of Microorganisms and Cell Cultures (DMSZ#12120). *Pseudomonas syringae* pv. *syringae* [26] was a kindly gift from M. Espinosa (EEZ-CSIC). The *Escherichia coli* strains DH5α [27], S17-1λpir [28], and CC118 [29], as well as *Rhizobium leguminosarum* bv. *trifolii* TT-7C [30], were also used. Plasmid pSEVA23PlexAGFPtir (pSEVA23GFP) that harbors a *gfp* gene under the control of the *PlexA* promoter and confers kanamycin resistance [31] was kindly provided by E. Martínez (CNB-CSIC, Spain). Seeds from *Oryza sativa* L. GLEVA and *Nicotiana tabacum* cv. Xanthi [32] plants were kindly provided by Castells Seeds Co. and F. Tenllado (CIB-CSIC), respectively.

### Media and growth conditions

MC medium was prepared as previously described [14]. The nitrogen-free MNF medium used was a modification of a previously described medium [33]: A Solution (g/900 ml): 0.8 g K_2_HPO_4_, 0.2 g KH_2_PO_4_, 0.1 g NaCl, 28 mg Na_2_FeEDTA, 25 mg Na_2_MoO_4_×2H_2_O, 100 mg yeast extract, pH 7.0; B Solution (g/100 ml): 0.2 g MgSO_4_×7H_2_O and 0.06 g CaCl_2_×2H_2_O. The two solutions were autoclaved separately and then the suitable carbon source, vitamins and trace elements solutions were added. For checking the utilization of insoluble phosphate, the MALP medium (MA modified medium with low phosphate) was used: (per liter) 0.2 g KH_2_PO_4_, 0.2 g Na_2_HPO_4_, 0.1 g MgSO_4_×7H_2_O, 0.1 g NH_4_Cl, 5 g calcium phosphate [34]. Once autoclaved, the CaCl_2_, vitamins and trace elements were added at the same concentrations than were used in the MC medium. VM-ethanol rich medium was prepared as previously described [35].

*E. coli* cells and *P. syringae* were grown at 37°C in lysogeny broth (LB) medium [36]. When required, *E. coli* was grown in VM-ethanol medium [37]. *Azoarcus* sp. CIB, *Azoarcus communis* and their derivatives were grown at 30°C either in MC medium, as previously described [14], or in VM-ethanol medium. *R. leguminosarum* was grown at 30°C in MC medium supplemented with 0.4% (w/v) glucose. When appropriate, kanamycin (50 µg ml^−1^) was added to the medium.

### Nitrogen fixation assays

Nitrogenase activity was measured by using the acetylene reduction activity assay for free-living bacteria [38]. *Azoarcus* sp. CIB cells were anaerobically grown in MC medium with 3 mM benzoate as sole source of carbon and energy until mid-exponential growth phase, and then they were pelleted by centrifugation at 5000×g for 15 min and resuspended in MNF medium, either in the presence or absence of ammonia. Cultures were grown microaerobically in closed batch cultures until they reached an *A*_600_ of 0.4. Then, 30 ml of the *Azoarcus* sp. CIB cultures were enclosed in 100 ml tubes sealed with rubber septa. Ten ml of air were removed and the same amount of acetylene was added (10% v/v). Gas samples were taken after 15 min, 1 h, 4 h, 24 h and 48 h of acetylene exposition at 25°C. Gas samples were analyzed for ethylene and acetylene content in a Perkin-Elmer 8310 gas chromatograph using nitrogen as the carrier gas and with a flow rate of 50 ml min^−1^ as described before [39].

### Inoculation of rice with *gfp*-expressing bacteria

Dehulled seeds of *O. sativa* GLEVA were surface sterilized by shaking at 25°C for 30 min in 30 ml of 1% (v/v) sodium hypochlorite. After three washes for 10 min in sterile water, the seeds were incubated in VM-ethanol for 48 hours, and only the uncontaminated seedlings were selected for inoculation. The germination continued on sterilized moist water filter paper for 24 h prior to inoculation with *gfp*-expressing bacterial cells. To obtain the *gfp*-expressing bacteria, we transferred by conjugation the pSEVA23GFP plasmid, that express the green fluorescent protein (GFP) under the *PlexA* promoter [40], from *E. coli* S17-1λ*pir* (pSEVA23GFP) to *Azoarcus* sp. CIB and *A. communis* strains using protocols previously described [14]. The selection of exconjugants was made as described before [14], and the expression of GFP was monitored by observation of bright green fluorescence cells under UV light. To inoculate the rice seedlings with the *gfp*-expressing bacteria, *Azoarcus* sp. CIB (pSEVA23GFP), *Azoarcus communis* strain SWub3 (pSEVA23GFP) and *E. coli* S17-1λpir (pSEVA23GFP) were grown in VM-ethanol medium at 30°C until they reached an *A*_600_ of 0.5. Cells were then harvested by centrifugation, washed with sterile 0.9% NaCl (w/v) solution, resuspended in sterile distilled water, and inoculated independently by pipetting 1 ml of the cell suspension onto the surface of the seedling in aseptic conditions. After the inoculation of the seedlings, plants were grown at 25°C with the natural daily light period (approximately 10 h of light and 14 of darkness) for 5–10 days.

### Quantification of *gfp*-expressing bacteria inside rice roots

Plants were sampled at 5 days after the inoculation of seedlings with the *gfp*-expressing bacteria. Loosely attached bacteria were removed by exhaustively washing the roots with sterile water. Then, the roots were surface sterilized by immersion for 3 min in 1% sodium hypochlorite solution. After three washes with sterile distilled water, the roots were homogenized using a sterile pestle and mortar, and the extracts diluted in 1 ml sterile saline solution (0.9% NaCl) as described before [1]. Serial dilutions of the extracts were plated onto VM-ethanol solid medium in the presence of kanamycin to determine the number of CFU containing the pSEVA23GFP plasmid. Moreover, the expression of GFP in the kanamycin-resistant recovered bacteria was confirmed by epifluorescence microscopy.

### Fluorescence microscopy studies

The root samples were collected 5 or 10 days after inoculation, washed with water, hand cut, mounted on a microscope slide and immediately examined under an inverted microscope Leica DMI6000B to visualize the *gfp*-labelled bacteria, or a Leica TCS-SP5-AOBS confocal laser microscope, on green channel, with excitation of 488 nm (laser beam) and emission of 500–547 nm to see the bacteria inside the plant tissues.

### Light microscopy and immunocytochemistry studies

Small samples of rice roots (0.5–1 cm) inoculated with *Azoarcus* sp. CIB for 7 days were cut with a razor blade and fixed in 2.5% glutaraldehyde in 0.05 M Na-cacodylate buffer (pH 7.4) containing 25 mg per ml sucrose [41], and vacuum-infiltrated to enhance penetration of the fixative. Dehydration was performed using an ethanol series and nodules pieces were infiltrated and finally embedded in LR White resin (London Resin Corporation), using gelatine capsules. The post-fixation with osmium tetroxide was not carried out in order to overcome the masking of antigenic sites with osmium. Two kinds of polymerization were performed: by heat at 60°C for 24 h (for light microscopy) or by ultraviolet light at −20°C for 48 h to avoid protein denaturalization (for immunocytochemistry). Semi-thin (1 µm) and ultrathin (70–80 nm) sections of each sample were cut with a Reicher Ultracut S Ultramicrotome (Vienna, Austria) fitted with a diamond knife. Semithin sections for light microscopy (LM) were collected on glass sides and stained with 1% (w/v) toluidine blue in aqueous sodium borate [42] and directly observed with a Zeiss Axiophot photomicroscope.

Immunolocalization was carried out using an antibody against the NifH subunit of the nitrogenase enzyme, kindly provided by Dr. Imperial (Centre for Plant Biotechnology and Genomics, UPM-INIA). Immunogold localisation was performed on 70 nm sections picked on pioloform-coated nickel grids as previously described [43], with some modifications. Grids were floated on TBS (10 mM Tris-HC1; 150 mM NaC1; 0.1% (v/v) Tween-20, pH 7.4), containing 20 mg of BSA per ml, for 1 h at 37°C. Sections were then incubated in primary antibody, anti NifH, diluted 1∶500 in the same buffer (TBS containing 20 mg of BSA per ml) for 2 h at 37°C. Grids were then washed (five times, 3 min) in TBS containing 2 mg of BSA per ml. Incubation in goat anti-rabbit IgG-gold conjugate (GAR 15 nm; Bio-Cell) diluted 1∶40 in TBS with 2 mg of BSA per ml was performed for 1 h at 37°C. Sections were then rinsed in TBS containing 20 mg of BSA per ml (five times, 3 min), in the same buffer containing 0.05% Triton X-100 (three times, 3 min), and finally in distilled water (3 min). Controls were performed without anti NifH antibody. Counterstaining of sections was obtained with 2% aqueous uranyl acetate and lead citrate [44] for 1 min. After rinsing and air-drying, the sections were observed with a STEM-LEO 910 microscope at 80 kV.

### Indoleacetic acid (IAA) production assay

Production of IAA was estimated by growing the bacterial strains at 30°C for 48 h in MC minimal medium supplemented with 2.5 mM *L*-tryptophan which acts as a precursor for IAA synthesis. IAA produced per milliliter of culture was estimated by mixing 14 ml of Salkowski reagent (12 g/l FeCl_3_ dissolved in 7.9 M H_2_SO_4_) [45] with 1 ml culture supernatants, followed by measuring absorbance at 530 nm wavelength after 30 min of incubation at 25°C [46]. A calibration curve for the estimation of the amount of IAA was made by using IAA as standard [47].

### Insoluble phosphate solubilisation assay

Bacterial cells were grown on MC medium until they reached mid exponential growth phase, then they were collected by centrifugation and washed three times with sterile saline solution. The cell pellet was resuspended in 0.5 ml of sterile MALP medium. A drop of 10 µl of the resuspended cell solution was deposited in a MALP solid media. The halo around the colonies was visualized after 7 days of incubation of plates at 30°C. The ability of the bacteria to solubilise insoluble phosphate was described by the halo formation as described before [48].

### Plant pathogenicity test

*Azoarcus* sp. CIB and *Pseudomonas syringae* pv. *syringae*, a plant pathogen used as control, were infiltrated into the intercellular spaces of an old leaf of an intact plant of *Nicotiana tabacum* at a dose of 10^7^ colony forming units (CFU) per leaf area as described previously [49].

### Sequence data analyses

The nucleotide sequence of the *nifH* gene from *Azoarcus* sp. CIB has been submitted to GenBank with Accession number KJ814970. Phylogenetic analysis was carried out by using the Phylogeny.fr program [50].

## Results and Discussion

### *Azoarcus* sp. CIB is a nitrogen fixing bacterium

As indicated in Introduction, *Azoarcus* sp. CIB strain is a free-living bacterium able to degrade a high number of aromatic compounds either in the presence or in the absence of oxygen [14], [21], [24]. The recent sequencing of the genome of *Azoarcus* sp. CIB revealed that its size (5.25 Mb) is significantly bigger than that of other well-characterized *Azoarcus* strains, such as the rice endophyte *Azoarcus* sp. BH72 (4.37 Mb) or the free-living aromatic biodegrader “*A. aromaticum*” EbN1 strain (4.72 Mb), indicating that the strain CIB contains a more complete genetic repertoire that might be used for the adaptation to different habitats. Since free-living *Azoarcus* strains have never been isolated from inside of living plants [34] and the plant-associated *Azoarcus* strains could not be isolated from root-free soil behaving as obligate endophytes [13], we explored the possibility that CIB strain may behave as a facultative endophyte, i.e., a bacterium that has a stage in its life cycle in which it exists outside host plants as a free-living bacterium [51]. Giving that certain plant-associated bacteria have the ability to fix nitrogen, solubilize minerals, and produce phytohormones [52]–[54], we tested whether *Azoarcus* sp. CIB showed some of these traits.

To investigate the nitrogen fixation ability of *Azoarcus* sp. CIB, we firstly cultivated bacterial cells in nitrogen-free MNF medium, and we observed that the CIB strain was able to grow, suggesting that it utilizes atmospheric nitrogen. In addition, nitrogenase activities of cells growing in MNF medium were one order of magnitude higher than those of cells grown in MNF medium supplemented with NH_4_Cl as nitrogen source (Table 1). Therefore, these data indicate that *Azoarcus* sp. CIB is a nitrogen fixing bacterium.

**Table 1:** Determination of *Azoarcus* sp. CIB nitrogenase activity.

	24 h		48 h	
	*µmol/cells (Abs_600_ 0.4)*	*µmol/g fresh culture*	*µmol/cells (Abs_600_ 0.4)*	*µmol/g fresh culture*
–N (30 ml)	1,37±0.4	13,69±2.1	2,41±0.3	24,03±4.7
+N (30 ml)	0,24±0.07	1,51±0.4	0,26±0.09	1,68±0.55

Total (µmol C_2_H_4_/30 ml of culture at *Abs_600_* of 0.4) and specific (µmol C_2_H_4 _g^−1^bacteria fresh weight 24 h^−1^ or 48 h^−1^) nitrogenase activity of *Azoarcus* CIB grown in MNF medium with 0.37 g/l NH_4_Cl (+N) or without ammonia (–N) as nitrogen source. The values are the average from three independent experiments +/− S. D.

*In silico* analysis of the unpublished draft of *Azoarcus* sp. CIB genome sequence identified a *nifH* gene whose product shows 76.4% amino acid sequence identity with the NifH subunit of the nitrogenase protein involved in nitrogen fixation in *Azoarcus* sp. strain BH72 [55]. A phylogenetic analysis of the *nifH* gene product from strain CIB revealed that it branched with that of other soil *Azoarcus* strains and some other β subclass of Proteobacteria (Figure S2). In contrast, NifH from *Azoarcus* sp. BH72 and other endophytic *Azoarcus* strains (aligned with the orthologs in *Azotobacter vinelandii*, *Pseudomonas stutzerii* and *Klebsiella oxytoca* of the γ subclass of Proteobacteria (Figure S2). Phylogenetic distances of NifH proteins indicate a possible lateral gene transfer of *nif* genes to *Azoarcus* from a common donor of the α Proteobacteria at the time of species diversification, with more recent and independent transfer events in some plant-associated *Azoarcus* species, such as the BH72 strain, forming a monophyletic unit with those of γ *Proteobacteria* [56]. The apparent divergence of the NifH protein in the *Azoarcus* genus was described previously where it was postulated the existence of two groups of *nifH* sequences that correspond to the soil and plant-associated *Azoarcus* strains, respectively [56].

### *Azoarcus* sp. CIB solubilises insoluble inorganic phosphate

Several reports showed the ability of different bacteria to solubilise insoluble inorganic phosphate compounds [57]. In fact, since most of the soils under cultivation contain insoluble phosphates, the ability of the bacteria associated with plants to solubilise precipitated phosphates or enhance phosphate availability to the plant represents a mechanism of plant growth promotion (PGP) under field conditions [58]. To test whether *Azoarcus* sp. CIB is able to grow in minimal medium using insoluble phosphate as the major phosphorous source, we used a plate assay that provides a semiquantitative estimation of the phosphate solubilisation ability. According to this method, clearing zones around the microbial colonies in media containing insoluble mineral phosphates (mostly tricalcium phosphate or hydroxyapatite) as single phosphorous source can be visualized [53]. After 14 days incubation, *Azoarcus* sp. CIB showed an apparent cell growth and a clear halo around the colonies (Figure S3), indicating that the CIB strain is able to use insoluble phosphorous.

### *Azoarcus* sp. CIB produces IAA

**Figure 1:**
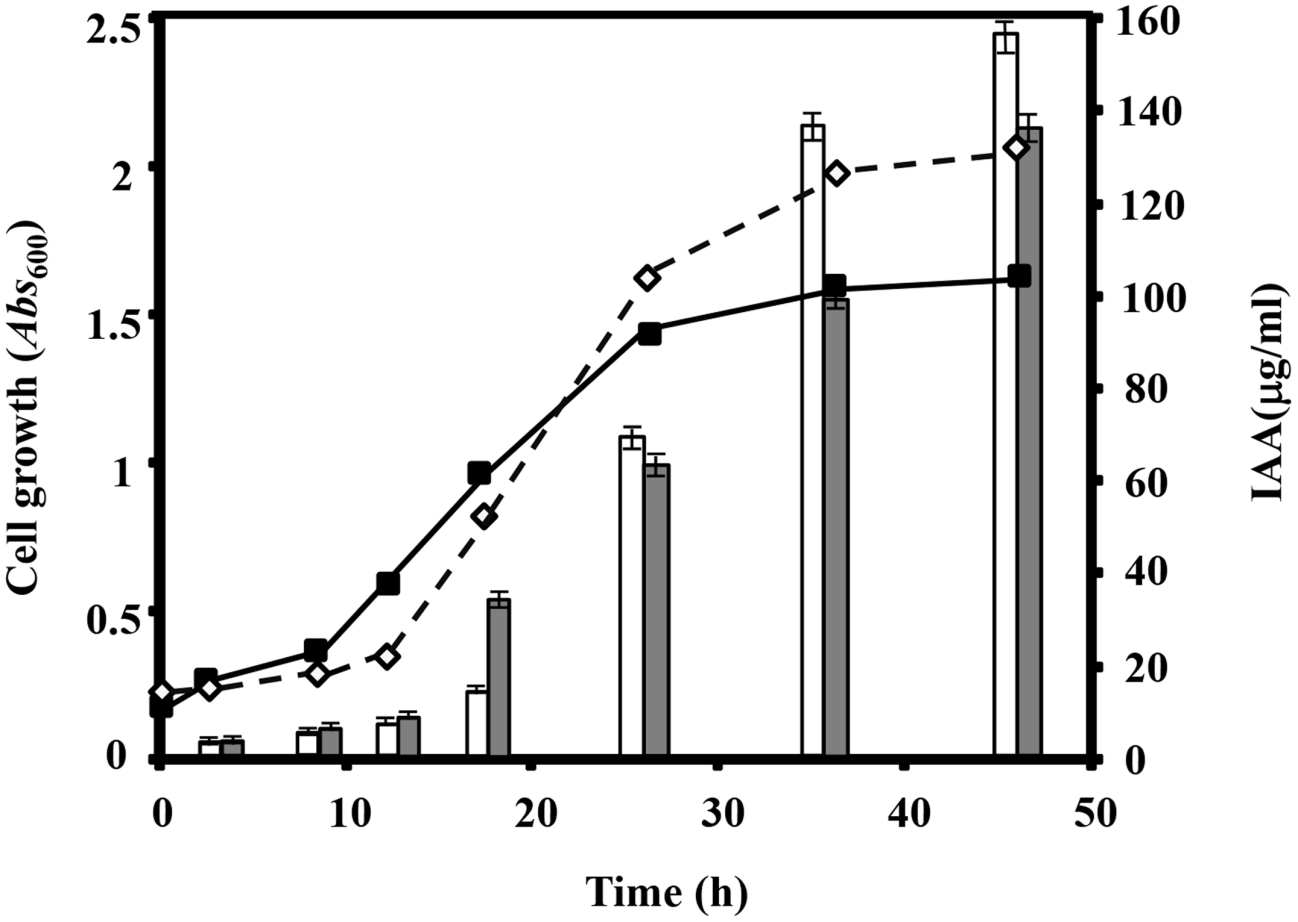
*Azoarcus* sp. CIB produces IAA. Production of IAA by *Azoarcus* sp. CIB (white bars) and R. *leguminosarum* bv. *trifolii* TT-7C (grey bars) along the growth curve. Growth of *Azoarcus* sp. CIB (black squares) and *R. leguminosarum* bv. *trifolii* TT-7C (grey rhombus) is also represented. IAA was quantified as detailed in Materials and Methods. Graphed values of are the average from three independent experiments +/− S. D.

The ability to produce the plant hormone IAA, a naturally occurring auxin, is widespread among microorganisms that are commonly associated with plant surfaces [59]. Microbes from the rhizosphere of different crops appear to have a greater potential to synthesize and release IAA as secondary metabolite because of the relatively rich supply of tryptophan [60]. IAA has been implicated in regulating a variety of developmental and cellular processes in plants such as cell extension, cell division, vascular differentiation, root formation, apical dominance, and tropism [61]. To analyze the ability of *Azoarcus* sp. CIB to produce IAA, cells were grown on MC medium supplemented with 2.5 mM tryptophan. Relative levels of IAA along the growth curve were estimated by a colorimetric assay (see details in Material and Methods). Maximal production of IAA was achieved after 48 h of growth (Figure 1). When tryptophan was not added to the medium, no colorimetric change was detected (data not shown). *Rhizobium leguminosarum* bv. *trifolii* TT-7C, a strain that is able to produce IAA [60], was used as a positive control and it showed levels of IAA production similar to those of *Azoarcus* sp. CIB, with a maximal production also after 48 h of growth (Figure 1). It is interesting to mention that there is an important increase of IAA production at the end of the exponential growth-phase (Figure 1), suggesting a phase-dependent production of IAA as it has been already reported in other bacteria and fungi strains [60], [62], [63]. Although that as far as we know, this is the first report of an *Azoarcus* strain able to produce IAA to understand the role of IAA production on the putative relationship between *Azoarcus* sp. CIB and plants, more experiments have to be done.

### *Azoarcus* sp. CIB does not cause plant pathogenic effects

**Figure 2:**
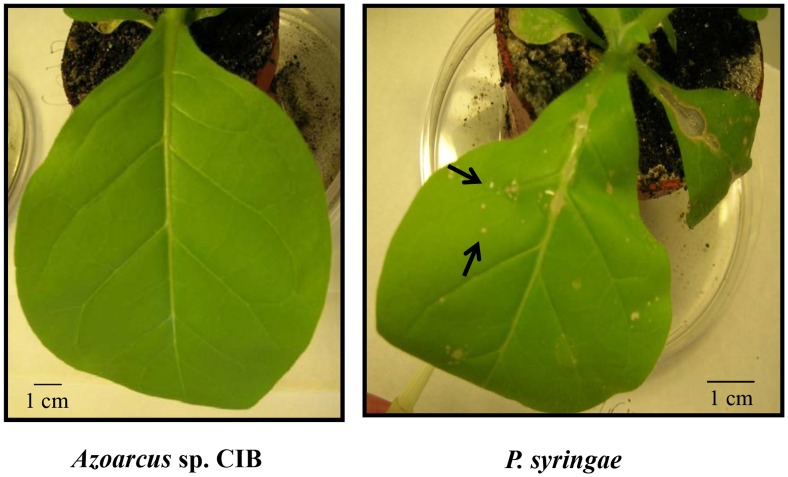
*Azoarcus* sp. CIB is a non-pathogenic bacterium. Pathogenicity test on tobacco leaves. Bacteria were infiltrated into the intercellular spaces of a tobacco leaf, which was inspected for a visible necrosis after 2 days. Necrosis is visible as light spots (arrows) on leaves infiltrated with *Pseudomonas syringae pv. syringae* (right) and not visible on leaves infiltrated with *Azoarcus* sp. CIB (left).

The results showed above reveal that *Azorcus* sp. CIB elicits properties of plant-associated bacteria such as the ability to fix nitrogen, produce IAA and solubilise insoluble phosphate, suggesting that the CIB strain could interact with plants. To check whether *Azoarcus* sp. CIB could cause defense reactions in a plant, a commonly used pathogenicity test [49] was done. Whereas the inoculation of *Azoarcus* sp. CIB did not elicit a hypersensitive response leading to visible necrosis in tobacco leaves within 10 days, leaves treated with the plant pathogen *P. syringae* pv. *syringae* developed this phenotype in two days (Figure 2). Moreover, no macroscopically visible disease symptoms were observed on rice seedlings infected with *Azoarcus* sp. CIB cells (data not shown). These results suggest that *Azoarcus* sp. CIB is not a plant phatogen, which is in agreement with a previous observation that demonstrated the non-pathogenic effect of some endophytic *Azoarcus* strains on tobacco and rice [64].

All these results showed that *Azorcus* sp. CIB is not a pathogen of plants that elicit some of the properties of the plant-associated bacteria such ability to fix nitrogen, to produce IAA and to solubilise insoluble phosphate. On behalf of that we next explored the ability of *Azoarcus* sp. CIB to associate with plants.

### *Azoarcus* sp. CIB interacts with rice roots as an endophyte

**Figure 3:**
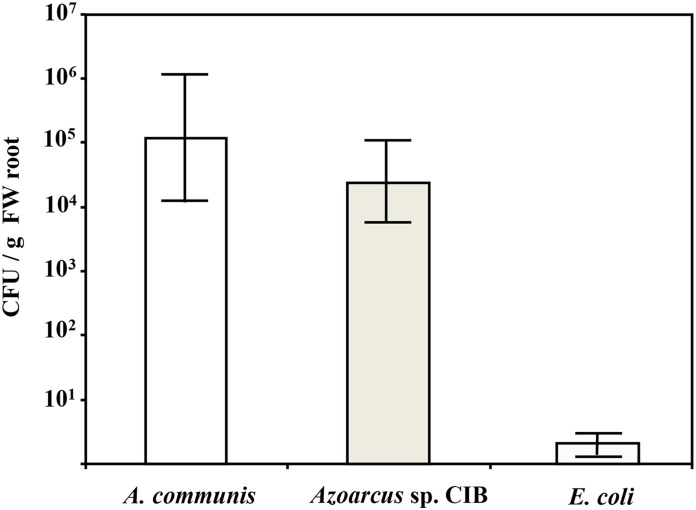
Quantification of the bacterial endophytic population within inoculated rice roots. Rice seedlings were inoculated with *Azoarcus communis* Swub3 (*A. communis*), *Azoarcus* sp. CIB (*Azoarcus* sp. CIB), and *E. coli* S17-1λ*pir* (*E. coli*) containing plasmid pSEVA23GFP) that expresses the *gfp* gene as indicated in Materials and Methods. Plants were grown at 25°C for 5 days, and the kanamycin-resistant bacteria within the root tissue were determined as detailed in Materials and Methods. Graphed values of CFU per g of fresh root (FW) are the average from three independent experiments +/− S. D.

Since *Azoarcus* sp. CIB shows typical traits of plant-associated bacteria (see above) and it is well-known that some *Azoarcus* strains are rice endophytes [2], it was tempting to speculate that the CIB strain could share also an endophytic lifestyle. To check whether *Azoarcus* sp. CIB is also an endophyte, we monitored the presence of CIB cells in the interior of rice roots inoculated with this bacterium. Since the *gfp* marker gene has been proved to be very useful in colonisation studies and to visualise plant endophytes [65], [66], we generated bacterial cells containing plasmid pSEVA23GFP that expresses the *gfp* reporter gene. Inoculation of rice seedlings with *Azoarcus* sp. CIB (pSEVA23GFP) or with *Azoarcus communis* SWub3 (pSEVA23GFP), a previously reported rice endophyte bacterium [25], led to the detection of about 6×10^4^ CFU.gr^−1^ root and 1×10^5^ CFU.gr^−1^ root, respectively, in the interior of the roots 5 days after inoculation (Figure 3). These values are similar to those previously reported for re-isolations of plant endophytes [67]. Moreover, following rice inoculation with *Azoarcus* sp. CIB (pSEVA23GFP), most of the kanamycin-resistant CFUs (>90%) detected were fluorescent under UV light in an epifluorescence stereomicroscope (data not shown), confirming that most of the endophytic bacteria isolated from the inner tissues of the roots corresponded to the inoculated *Azoarcus* strains. In contrast, an insignificant number of CFUs (<10) of *E. coli* S17-1λpir (pSEVA23GFP) was isolated from inoculated rice seedlings. In summary, these results suggest that *Azoarcus* sp. CIB behaves as rice endophytic bacterium. Nevertheless, it is known that bacteria closely attached to crevices and/or embedded in mucilage might escape chemical surface sterilization of roots and behave as false endophytes. Therefore, to confirm that *Azoarcus* sp. CIB behaves as a true rice endophyte an *in planta* visualization of the bacterium should be accomplished by microscopy studies [2].

### *In planta* visualization of the endophytic lifestyle of *Azoarcus* sp. CIB

**Figure 4:**
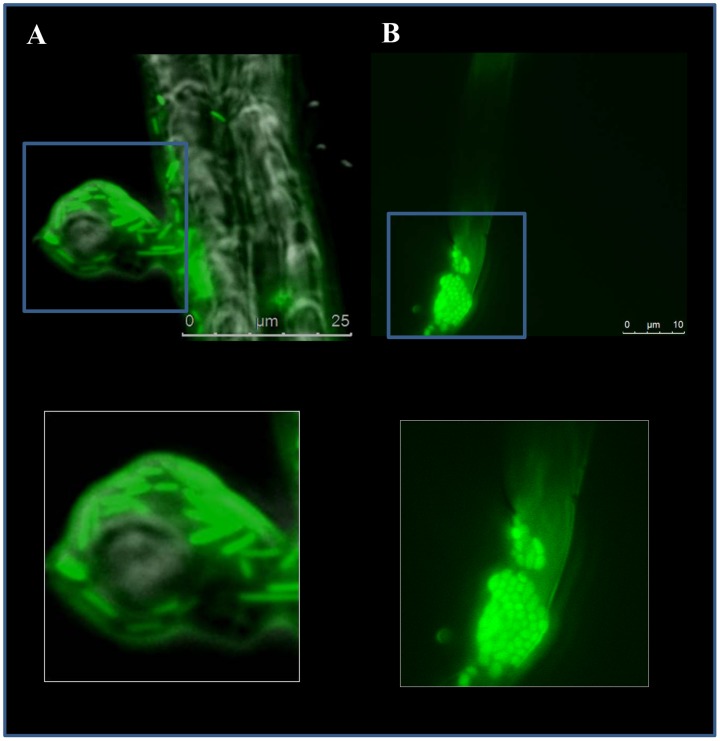
Images of rice roots inoculated with *Azoarcus* sp. CIB. Epifluorescence microscopy images of rice roots after 5 days (A) or 10 days (B) of inoculation with *Azoarcus* sp. CIB (pSEVA23GFP). A detail of each image is included (lower panels) to illustrate the morphological change of the cells.

By using epifluorescence microscopy, *Azoarcus* sp. CIB (pSEVA23GFP) cells expressing GFP were visible inside sterilised roots obtained 5 days (Figure 4A) and 10 days (Figure 4B) after the inoculation of the corresponding seedlings. The *gfp*-expressing CIB cells were particularly abundant just beneath the epidermal surface at the area of growing hairs (Figure 4A). Interestingly, there was a noticeable change in the cellular morphology of the bacteria at long colonization times. Hence, *Azoarcus* sp. CIB cells appeared to be shorter and more spherical after 10 days (Figure 4B) than after 5 days (Figure 4A) of inoculation. These changes in cellular shape are dependent on environmental conditions and have been reported previously in other plant-associated bacteria [68], [69]. In fact, it has been proposed that bacterial cells are better nourished upon successful colonisation, and cell shape is related to the growth rates within a particular environment [66].

**Figure 5:**
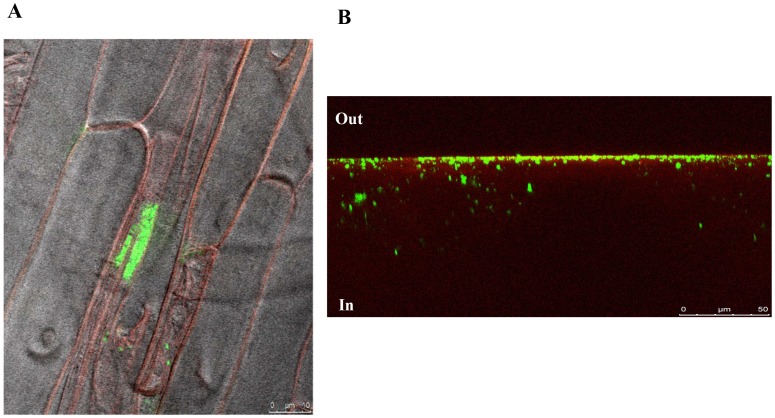
Colonization of rice roots by *Azoarcus* sp. CIB observed by confocal microscopy. Confocal images of rice roots after seven days colonization with *Azoarcus* sp. CIB (pSEVA23GFP) cells. (A) Bacteria are observed as single cells or as clusters of cells attached to the inner intercellular space of the exodermis. (B) XYZ projection of bacterial cells distributed on the inner part (In) or the surface (Out) of the rice root.

Internal colonization of rice roots by *Azoarcus* sp. CIB was confirmed by using confocal microscopy after 7 days of colonization (Figure 5). Internal distribution of cells showed a diverse pattern since bacteria could be either as single cells or cell clusters. *Azoarcus* sp. CIB (pSEVA23GFP) cells were mainly detected in the intercellular spaces of the exodermis (Figure 5A). A xyz projection of the image obtained from roots rice inoculated with *Azoarcus* sp. CIB (pSEVA23GFP) showed that bacteria distribute also in the inner parts of the exodermis (Figure 5B).

**Figure 6:**
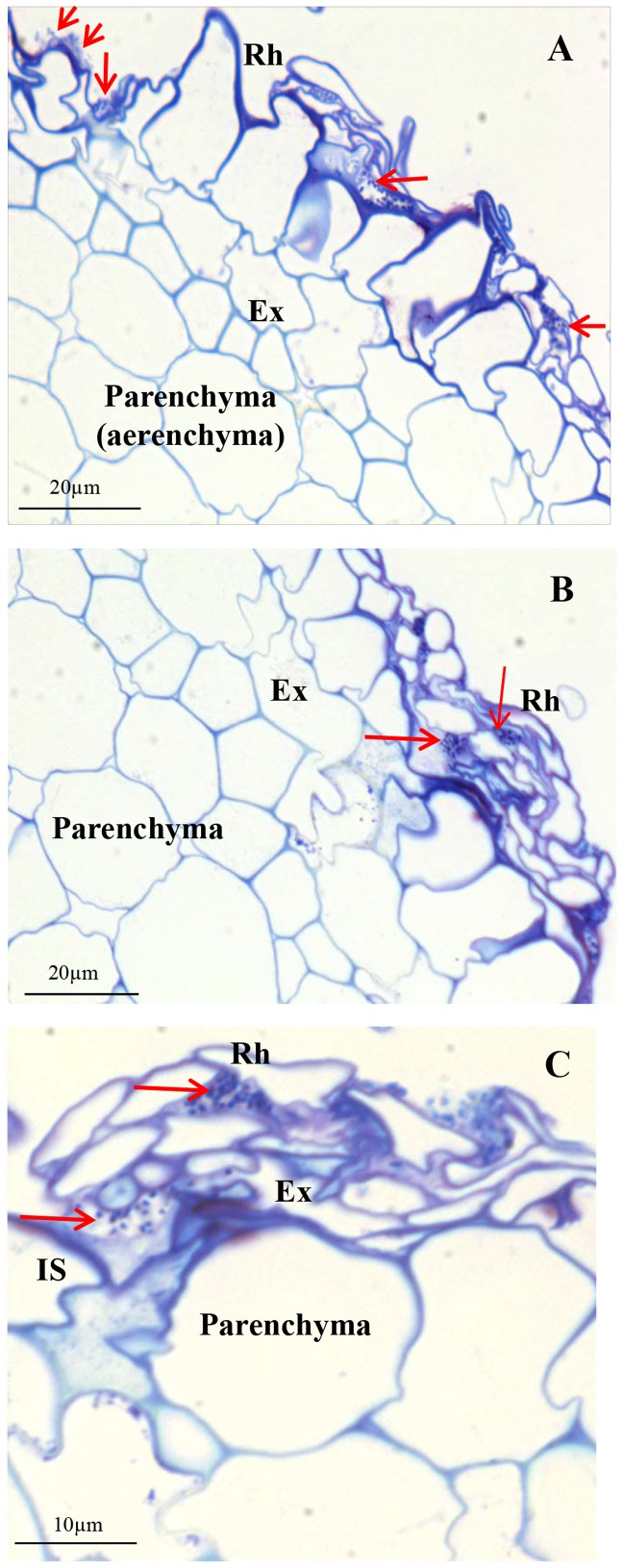
Light micrographs of transversal sections of rice roots inoculated with *Azoarcus* sp. CIB (pSEVA23GFP) cells and incubated for 7 days. Colonization of root surface and intercellular spaces under rhizodermis and the first layer of exodermis (A); intercellular colonization of the second and third layers of the exodermis (B); intercellular colonization of the deeper layer of the exodermis, just in contact with the parenchyma (C). IS, intercellular space; Ex, exodermis, Rh, rhizodermis. Red arrows indicate the localization of the bacterial cells.

**Figure 7:**
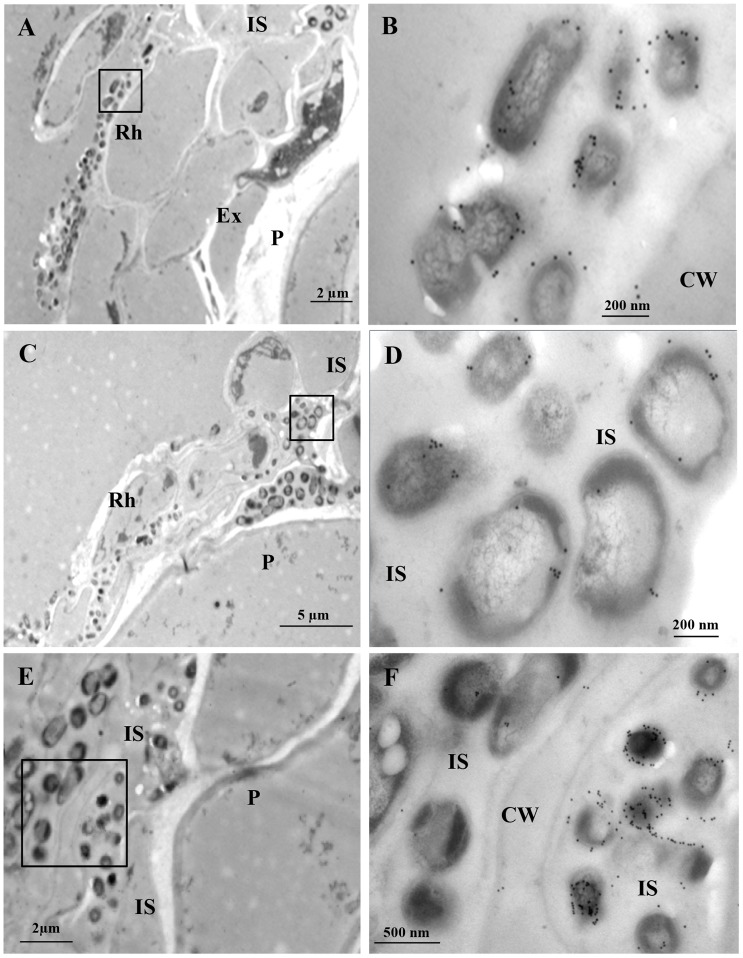
Electron microscopy observation of rice roots colonized by *Azoarcus* sp. CIB. Electron micrographs showing immunogold localization of NifH epitopes in rice roots inoculated with *Azoarcus* sp. CIB (pSEVA23GFP) cells for 7 days. Surface colonization (A), intercellular colonization of the second and third layers of the exodermis (C), intercellular colonization of the deeper layer of the exodermis, just in contact with the parenchyma (E). CW, cell wall; Ex, exodermis; IS intercellular space; Rh, rhizodermis; P, parenchyma. In order to distinguish gold particles, the framed areas in A, C and E are magnified in panels B, D and F, respectively.

To see in more detail how the *Azoarcus* sp. CIB cells locate in the internal part of the rice root, we prepared resin-embedded roots of rice seedlings grown axenically with *Azoarcus* sp. CIB (pSEVA23GFP) cells. Light and electron microscopy examination of theses samples confirmed that bacteria colonize the exodermis of roots. Thus, bacteria moved from the surface to intercellular spaces of rhizodermis and the second and third layer of the exodermis (Figure 6A and 6B), until they reached the deeper layer of the exodermis, just in contact with the parenchyma of the roots (Figure 6C). No bacteria were observed in rice roots that were not inoculated with *Azoarcus* sp. CIB (pSEVA23GFP) (data not shown), which confirms that the observed bacteria correspond to *Azoarcus* sp. CIB cells. Immunogold labelling of the ultraviolet ligh-polymerized samples with antiserum raised against the NifH protein and electron transmission microscopy confirmed the superficial (Figure 7A and 7B) and intercellular spaces colonization of all the layers of exodermis (Figure 7C to 7F). Labelling of NifH protein was specifically confined to the *Azoarcus* sp. CIB (pSEVA23) bacteria, although unspecific and very scarce gold particles were observed in the matrix of the intercellular spaces and in the cell wall of exodermis and parenchyma cells of rice roots. Gold particles were never observed in the cytoplasm or organelles of plant cells. Although gold particles can be visualized in *Azoarcus* sp. CIB (pSEVA23GFP) cells colonizing surface and first and second layer of exodermis, a higher amount of gold particles were detected in bacterial cells that colonize the deepest layers of exodermis (Figure 7E to 7F). This observation may reflect the fact that nitrogen fixation is an anaerobic process and bacteria that occupy the deeper layers of the exodermis have lower oxygen availability, hence being able to express abundantly the nitrogenase protein.

In summary, all the three different microscopy techniques used, i.e., laser confocal, optical and electron microscopy, allow us to conclude that the CIB bacterial strain is a new rice endophyte from the *Azoarcus* genus.

## Conclusions

This is the first report of a bacterium, *Azoarcus* sp. CIB, that was shown to degrade anaerobically a high number of aromatic compounds [14], [21], [24] and displays the ability to colonize two different ecological niches, i.e., soil and water as free-living bacteria and the inner tissues of the rice roots as a facultative endophyte. The previous thought that the *Azoarcus* genus comprises bacteria that fit into one of two major eco-physiological groups, i.e., either the free-living anaerobic biodegraders of aromatic compounds or the obligate endophytes unable to degrade aromatics under anaerobic conditions [9], [20], should be now revisited. *Azoarcus* sp. CIB may represent the prototype of a subgroup of *Azoarcus* strains that share the anaerobic biodegradation of aromatic hydrocarbons with an endophytic lifestyle. On the other hand, there is another subgroup of *Azoarcus*, whose prototype is the EbN1 strain [20], that degrade aromatics under anaerobic conditions but are unable to interact with plants. Phylogenetic analyses based on the 16S rDNA and *nifH* genes confirmed that obligate endophytes such as *Azoarcus* sp. BH72, *A. communis*, and *A. indigens*, and facultative endophytes, such as *Azoarcus* sp. CIB, belong indeed to different evolutionary branches.

*Azoarcus* sp. CIB shows also some traits of PGP bacteria such the ability to uptake insoluble phosphorous, production of IAA or nitrogen fixation, which makes this endophyte as a potential candidate for a more sustainable agricultural practice [70]. In addition, since *Azoarcus* sp. CIB is able to degrade, both aerobically and anaerobically, toxic aromatic compounds [14], the use of this bacterium in association with plants could offer an efficient, economic and sustainable phytoremediation technology [71].

## Supporting Information

Figure S1 **Neighbor-joining phylogenetic tree of** ***Azoarcus*** **bacteria based on the comparison of their 16SrRNA genes.** The branch length is proportional to the number of substitutions per site. The sequence of the 16SrRNA genes from *Azoarcus* sp. strain EC1–7 (EU708505), *Azoarcus* sp. strain EC2–7 (EU708500), *Azoarcus* sp. strain HxN1 (AF331975), *Azoarcus buckelii* strain U120 (AJ315676), *Azoarcus* sp. strain EbN1 (X83531), *Azoarcus anaerobius* strain DSM12081 (Y14701), *Azoarcus* sp. strain GYP_24 (JX981924), *Azoarcus taiwanensis* strain NSC3 (GQ389714), *Azoarcus* sp. strain C3b_A2 (JX575077), *Azoarcus tolulyticus* strain Tol-4 (NR_037058), *Azoarcus denitrificans* (U82665), *Azoarcus toluclasticus* strain MF63 (NR_024970), *Azoarcus* sp. strain KH32C (NC_020516), *Azoarcus* sp. strain T (AF129465), *Azoarcus* sp. strain CIB (AF515816), *Azoarcus* sp. strain DAO1 (DQ336177), *Azoarcus evansii* strain KB740 (NR_029266), *Azoarcus toluvorans* strain Td-21 (NR_025915), *Azoarcus* sp. strain S5b2 (L15532), *Azoarcus communis* strain SWub3 (NR_024850), *Azoarcus indigens* strain. VB32 (NR_024851), and *Azoarcus* sp. strain BH72 (NR_074801) were included in the analysis. The previously described as free-living *Azoarcus* are dashed in orange color and the plant-associated *Azoarcus* are dashed in green.
(TIF)

Figure S2 **Neighbor-joining phylogenetic tree based on the NifH protein sequence.** The bacteria included in the analysis are: *Azotobacter vinelandii* (YP_00297378.1), *Pseudomonas stutzeri* (YP_001171863.1), *Klebsiella oxytoca* (YP_005020938.1), *Azoarcus* sp. BH72 (YP_932042), *Oscillatoria* sp. (WP_007356926.1), *Azospirillum brasilense* Sp245 (YP_005030951.1), *Rhodopseudomonas palustris* CGA009 (NP_949954.1), *Rhodobacter capsulatus* (AAA26140.1), *Magnetospirillum magneticum* AMB-1 (YP_420937.1), *Rhizobium leguminosarum* (WP_0035927442.1), *Rhizobium etli* (NP_659736.1), *Mesorhizobium* sp. STM4661 (WP_006331760), *Sinorhizobium meliloti* (WP_018097454.1), *Leptospirillum ferrooxidans* (AF097517.1), *Azoarcus* sp. KH32C (YP_00550106.1), *Azoarcus* sp. CIB (KJ814970), *Azoarcus toluclasticus* MF63 (WP_018989049.1), *Bradyrhizobium japonicum* (WP_018319598.1), *Xanthobacter autotrophicus* Py2 (YP_001415004.1), *Azorhizobium caulinodans* ORS571 (YP_001526359.1), *Burkolderia xenovorans* LB400 (YP_553849.1), *Cupriavidus* sp. WS (WP_020202091.1) and *Herbaspirillum frisingense* (WP_006463090.1). The branch length is proportional to the number of substitutions per site. The position of *Azoarcus* sp. CIB is indicated by a red arrow.
(TIF)

Figure S3 **Solubilisation of the low soluble inorganic phosphate by** ***Azoarcus*** **sp. CIB.** The halos were observed after 7 days of incubation on solid MALP medium at 30°C.
(TIF)
