# Correction: *Azoarcus* sp. CIB, an Anaerobic Biodegrader of Aromatic Compounds Shows an Endophytic Lifestyle

**DOI:** 10.1371/journal.pone.0114955

**Published:** 2014-11-26

**Authors:** The PLOS ONE Staff

The first author’s name is mispelled. The correct name is: Helga Fernández-Llamosas.

The correct citation is: Fernández-Llamosas H, Prandoni N, Fernández-Pascual M, Fajardo S, Morcillo C, et al. (2014) *Azoarcus* sp. CIB, an Anaerobic Biodegrader of Aromatic Compounds Shows an Endophytic Lifestyle. PLoS ONE 9(10): e110771. doi:10.1371/journal.pone.0110771
