# Supplementary figures and images for: Azoarcus sp. CIB, an Anaerobic Biodegrader of Aromatic Compounds Shows an Endophytic Lifestyle
Source: PLoS One. 2014 Oct 23;9(10):e110771. doi: 10.1371/journal.pone.0110771 (PMC4207700; doi:10.1371/journal.pone.0110771)

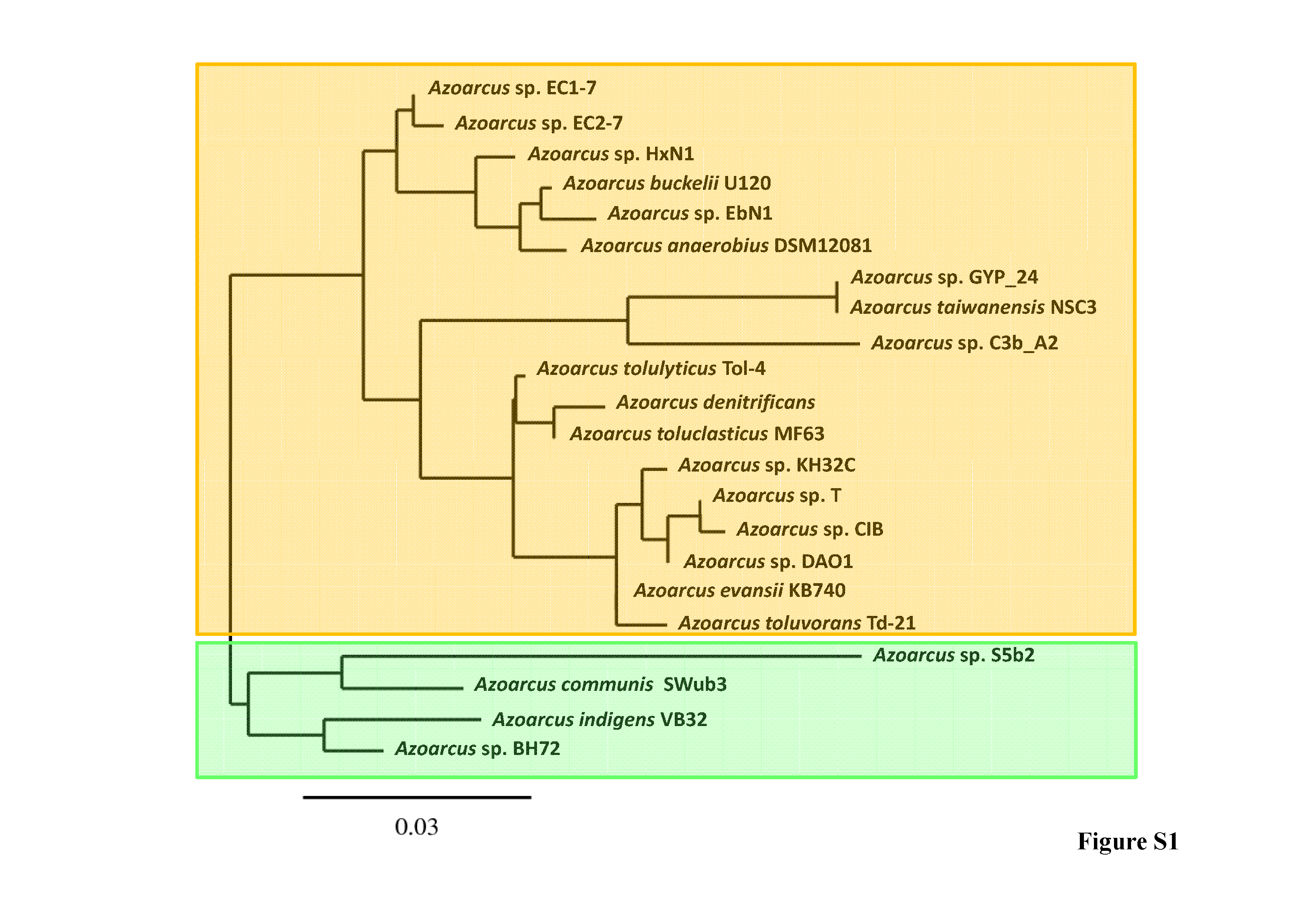

Supplement: Figure S1 — Neighbor-joining phylogenetic tree of Azoarcus bacteria based on the comparison of their 16SrRNA genes. The branch length is proportional to the number of substitutions per site. The sequence of the 16SrRNA genes from Azoarcus sp. strain EC1–7 (EU708505), Azoarcus sp. strain EC2–7 (EU708500), Azoarcus sp. strain HxN1 (AF331975), Azoarcus buckelii strain U120 (AJ315676), Azoarcus sp. strain EbN1 (X83531), Azoarcus anaerobius strain DSM12081 (Y14701), Azoarcus sp. strain GYP_24 (JX981924), Azoarcus taiwanensis strain NSC3 (GQ389714), Azoarcus sp. strain C3b_A2 (JX575077), Azoarcus tolulyticus strain Tol-4 (NR_037058), Azoarcus denitrificans (U82665), Azoarcus toluclasticus strain MF63 (NR_024970), Azoarcus sp. strain KH32C (NC_020516), Azoarcus sp. strain T (AF129465), Azoarcus sp. strain CIB (AF515816), Azoarcus sp. strain DAO1 (DQ336177), Azoarcus evansii strain KB740 (NR_029266), Azoarcus toluvorans strain Td-21 (NR_025915), Azoarcus sp. strain S5b2 (L15532), Azoarcus communis strain SWub3 (NR_024850), Azoarcus indigens strain. VB32 (NR_024851), and Azoarcus sp. strain BH72 (NR_074801) were included in the analysis. The previously described as free-living Azoarcus are dashed in orange color and the plant-associated Azoarcus are dashed in green. (TIF) [file pone.0110771.s001.tif]

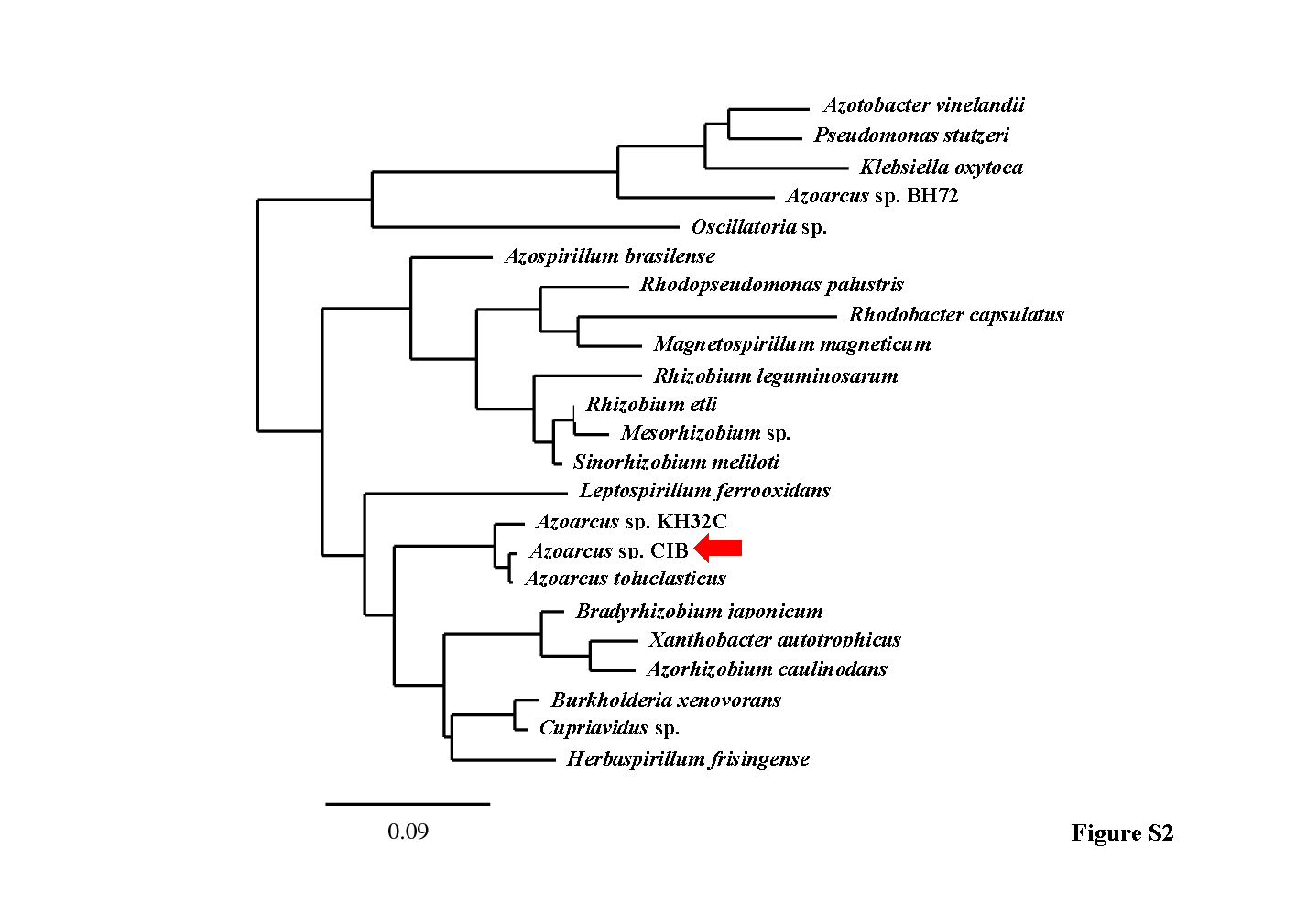

Supplement: Figure S2 — Neighbor-joining phylogenetic tree based on the NifH protein sequence. The bacteria included in the analysis are: Azotobacter vinelandii (YP_00297378.1), Pseudomonas stutzeri (YP_001171863.1), Klebsiella oxytoca (YP_005020938.1), Azoarcus sp. BH72 (YP_932042), Oscillatoria sp. (WP_007356926.1), Azospirillum brasilense Sp245 (YP_005030951.1), Rhodopseudomonas palustris CGA009 (NP_949954.1), Rhodobacter capsulatus (AAA26140.1), Magnetospirillum magneticum AMB-1 (YP_420937.1), Rhizobium leguminosarum (WP_0035927442.1), Rhizobium etli (NP_659736.1), Mesorhizobium sp. STM4661 (WP_006331760), Sinorhizobium meliloti (WP_018097454.1), Leptospirillum ferrooxidans (AF097517.1), Azoarcus sp. KH32C (YP_00550106.1), Azoarcus sp. CIB (KJ814970), Azoarcus toluclasticus MF63 (WP_018989049.1), Bradyrhizobium japonicum (WP_018319598.1), Xanthobacter autotrophicus Py2 (YP_001415004.1), Azorhizobium caulinodans ORS571 (YP_001526359.1), Burkolderia xenovorans LB400 (YP_553849.1), Cupriavidus sp. WS (WP_020202091.1) and Herbaspirillum frisingense (WP_006463090.1). The branch length is proportional to the number of substitutions per site. The position of Azoarcus sp. CIB is indicated by a red arrow. (TIF) [file pone.0110771.s002.tif]

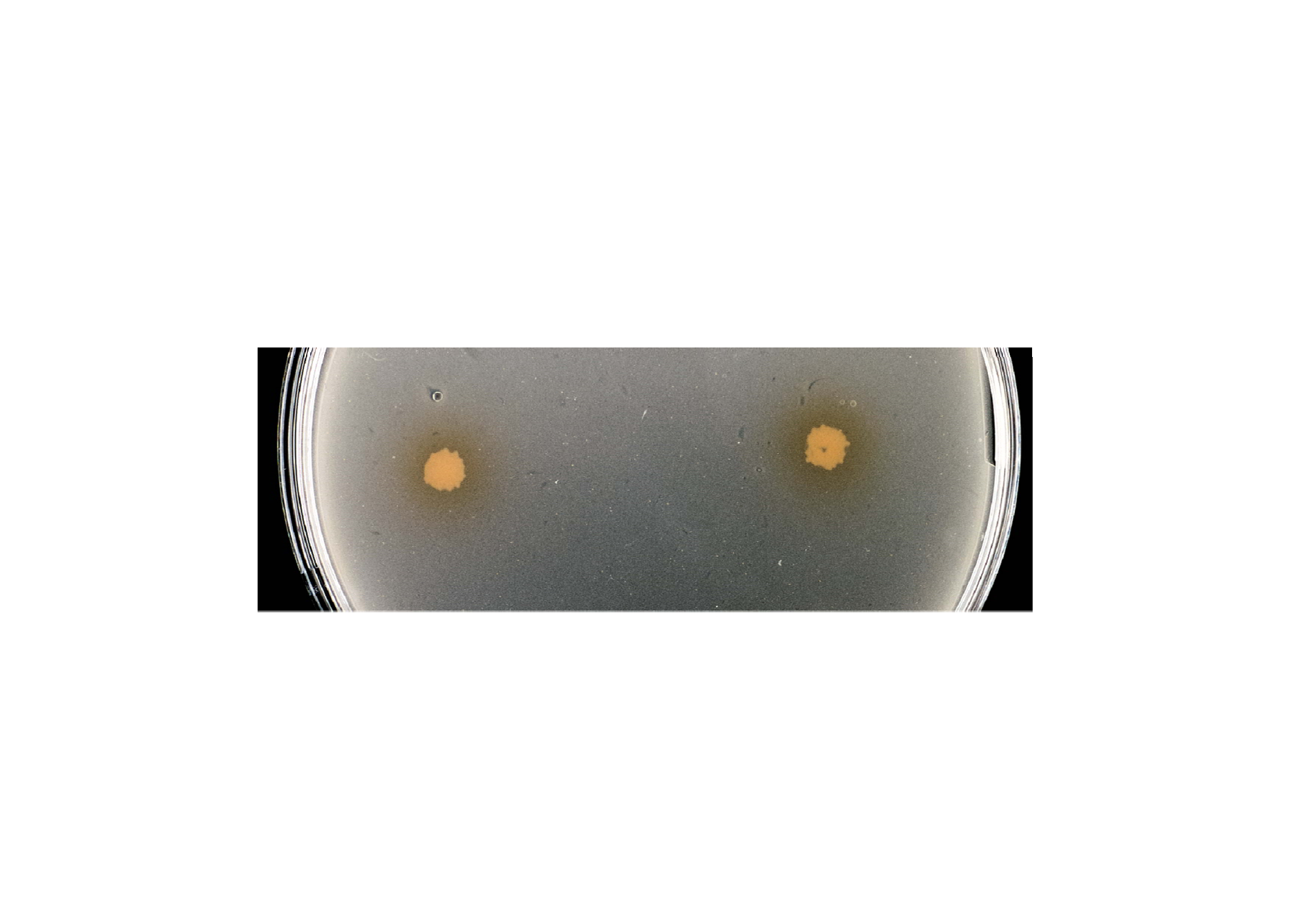

Supplement: Figure S3 — Solubilisation of the low soluble inorganic phosphate by Azoarcus sp. CIB. The halos were observed after 7 days of incubation on solid MALP medium at 30°C. (TIF) [file pone.0110771.s003.tif]
